# 1849. Prevalence, Phenotypic and Genotypic Characteristics, and Clinical Presentations of *Staphylococcus argenteus* Causing Bloodstream Infections in United States Hospitals

**DOI:** 10.1093/ofid/ofac492.1478

**Published:** 2022-12-15

**Authors:** Rafael Mendes, Lalitagauri M Deshpande, Cecilia G Carvalhaes, Drew Bell, Sue Kehl, Audrey Wanger, Mariana Castanheira, Rodrigo E Mendes

**Affiliations:** Univille University, Joinville, Santa Catarina, Brazil; JMI Laboratories, North Liberty, Iowa; JMI Laboratories, North Liberty, Iowa; Seattle Children's Hospital, Seattle, Washington; Medical College of Wisconsin, Milwaukee, Wisconsin; McGovern Medical School, Houston, Texas; JMI Laboratories, North Liberty, Iowa; JMI Laboratories, North Liberty, Iowa

## Abstract

**Background:**

*Staphylococcus argenteus* (SAR) is a novel species within the *S. aureus* (SAU) complex. SAR has been misidentified as SAU but has been increasingly reported worldwide as a pathogen. This study evaluated the prevalence of SAR causing bloodstream infection (BSI) in US centers, and the phenotypic, genotypic, and clinical outcomes associated with SAR BSI.

**Methods:**

785 SAU (326/459 MRSA/MSSA) from blood cultures (BC) of patients in 31 sites located in all 9 US Census Divisions during the SENTRY Antimicrobial Surveillance Program for 2019 were included. Isolates were screened for SAR by multiplex PCR. Isolates were subjected to MALDI-TOF and susceptibility (S) testing using the CLSI method. Isolates were subjected to genome sequencing, followed by DNA analysis.

**Results:**

0.4% (3/785) SAR were detected and originated from Milwaukee (12 yo female; patient 1), Houston (64 yo male; patient 2), and Seattle (21 yo male; patient 3). Patient 1 had a SAR recovered from BC 10 days after admission, but additional clinical information was not available. Patient 2 had end-stage renal disease and was admitted due to MRSA bacteremia/endocarditis, along with septic embolism in the lungs and brain. SAR grew from BC on day 4 and vancomycin was prescribed; the patient died on day 24. Patient 3 presented fever and chills in the 24 hours pre-admission, with a medical history significant for catheter-associated BSI. SAR was cultured from BC upon admission, and the patient received linezolid (5 days), but BCs remained positive. The central line was removed, and BCs cleared thereafter. MALDI-TOF generated highest scores for SAR for all 3 strains, but scores ≥2.0 were also obtained for SAU and *S. schweitzeri*. SAR (ST2198) from patient 1 was S to all agents tested, as were the other 2 strains (ST2250 and ST1223), except for oxacillin. The latter 2 strains carried SCC*mec* type IV(2B) and a *dfrG* was also detected in the ST2250 lineage strain. All 3 strains carried multiple virulence genes.

**Conclusion:**

Low prevalence of SAR causing BSI was observed in US hospitals in 2019; however, SAR can clearly cause invasive infections. ST2250 has predominantly been detected in the USA and Canada, but this study showed distinct lineages causing BSI, including strains carrying *mecA* and various virulence genes.

**Disclosures:**

**Rafael Mendes, BS**, JMI Laboratory: Grant/Research Support **Lalitagauri M. Deshpande, PhD**, Melinta: Grant/Research Support|Pfizer: Grant/Research Support **Cecilia G. Carvalhaes, MD, PhD**, AbbVie: Grant/Research Support|Cidara: Grant/Research Support|Melinta: Grant/Research Support|Pfizer: Grant/Research Support **Mariana Castanheira, PhD**, AbbVie: Grant/Research Support|Cidara: Grant/Research Support|GSK: Grant/Research Support|Melinta: Grant/Research Support|Pfizer: Grant/Research Support|Shionogi: Grant/Research Support **Rodrigo E. Mendes, PhD**, AbbVie: Grant/Research Support|Cidara: Grant/Research Support|GSK: Grant/Research Support|Melinta: Grant/Research Support|Nabriva Therapeutics: Grant/Research Support|Office for Assistant Secretary of Defense for Health Affairs: Grant/Research Support|Pfizer: Grant/Research Support|Shionogi: Grant/Research Support|Spero Therapeutics: Grant/Research Support.

